# Factors Influencing Spatial Variability in Nitrogen Processing in Nitrogen-Saturated Soils

**DOI:** 10.1100/tsw.2001.96

**Published:** 2001-10-17

**Authors:** Frank S. Gilliam, Frank C.C. Somerville, Frank N.L. Lyttle, Frank M.B. Adams

**Affiliations:** Department of Biological Sciences, Marshall University, Huntington, WV 25755-2510, USA

**Keywords:** base cations, hardwood forests, nitrogen saturation, nitrogen mineralization, nitrification, microbial ecology, amoA gene amplification

## Abstract

Nitrogen (N) saturation is an environmental concern for forests in the eastern U.S. Although several watersheds of the Fernow Experimental Forest (FEF), West Virginia exhibit symptoms of N saturation, many watersheds display a high degree of spatial variability in soil N processing. This study examined the effects of temperature on net N mineralization and nitrification in N-saturated soils from FEF, and how these effects varied between high N-processing vs. low N-processing soils collected from two watersheds, WS3 (fertilized with [NH_4_]_2_SO_4_) and WS4 (untreated control). Samples of forest floor material (O1 horizon) and mineral soil (to a 5-cm depth) were taken from three subplots within each of four plots that represented the extremes of highest and lowest rates of net N mineralization and nitrification (hereafter, high N and low N, respectively) of untreated WS4 and N-treated WS3: control/low N, control/high N, N-treated/low N, N-treated/high N. Forest floor material was analyzed for carbon (C), lignin, and N. Subsamples of mineral soil were extracted immediately with 1 N KCl and analyzed for NH4+ and NO_3_ to determine preincubation levels. Extracts were also analyzed for Mg, Ca, Al, and pH. To test the hypothesis that the lack of net nitrification observed in field incubations on the untreated/low N plot was the result of absence of nitrifier populations, we characterized the bacterial community involved in N cycling by amplification of amoA genes. Remaining soil was incubated for 28 d at three temperatures (10, 20, and 30°C), followed by 1 NKCl extraction and analysis for NH_4_ and NO_3_. Net nitrification was essentially 100% of net N mineralization for all samples combined. Nitrification rates from lab incubations at all temperatures supported earlier observations based on field incubations. At 30°C, rates from N-treated/high N were three times those of N-treated/low N. Highest rates were found for untreated/high N (two times greater than those of N-treated/high N), whereas untreated/low N exhibited no net nitrification. However, soils exhibiting no net nitrification tested positive for presence of nitrifying bacteria, causing us to reject our initial hypothesis. We hypothesize that nitrifier populations in such soil are being inhibited by a combination of low Ca to Al ratios in mineral soil and allelopathic interactions with mycorrhizae of ericaceous species in the herbaceous layer.

## INTRODUCTION

The cycling of nitrogen (N) in soils of terrestrial ecosystems is controlled by processes that often are quite spatially heterogeneous. Such heterogeneity can be important in determining the response of ecosystems to disturbances such as forest harvesting and excess inputs of N to forests via atmospheric deposition. For example, whereas spatially discrete areas of high N mineralization rates in soils ("hot spots") may serve as the source of N loss following disturbance, similar areas of low net N mineralization as a result of high immobilization of N may serve as an N sink, thereby mitigating the ecosystem-level loss of N. Walley et al.[1] found that landscape variability in N mineralization and nitrification was closely related to patterns of disturbance responses in a Canadian boreal forest. Morris and Boerner[2,3] found substantial landscape-scale variability in patterns of nitrification and microbial biomass in eastern hardwood forest soils, and concluded that moisture was a major factor in determining such variability. Some studies have found spatial variability of N processing in forest soils to be related to gap formation and leaf litter chemistry of contrasting tree species[4], whereas other studies have stressed hydrologic regime as a source of spatial patterns in soil N processing[5].

The Watershed Acidification Study was initiated by the U.S. Department of Agriculture Forest Service in 1988 at the Fernow Experimental Forest (FEF), Parsons, WV to examine ecosystem responses to aerial applications of $(NH_4)_2SO_4$ to an entire watershed (WS3), including response of soils and herbaceous-layer plants[6] and changes in stream chemistry[7]. Evidence from these and other studies indicates that untreated watersheds of FEF already have become N saturated from high ambient levels of atmospheric deposition of N[7,8]. However, spatial patterns of $NO_3^-$ in soil water of one untreated watershed (WS4) suggest that, although the watershed shows general signs of N saturation, microenvironmental variability (e.g., soil factors) may greatly limit rates of N processing in some areas[9]. Gilliam et al.[9] demonstrated that these areas of limited rates of N mineralization and nitrification are associated with high cover of ericaceous species (i.e., *Vaccinium* spp.) and suggested that N-processing microbes in these soils may be inhibited by the allelopathic effects of ericoid mycorrhizae. An additional explanation for the lack of net nitrification in soils within this otherwise N-saturated watershed is that nitrifier populations are essentially absent in soils exhibiting negligible nitrification.

The purpose of this study was to investigate the relationship between microbial communities and rates of N mineralization and nitrification in soils of N-treated WS3 and untreated WS4. In addition, this study focused on soils within each watershed that have exhibited the extremes (high vs. low) in rates of N processing to investigate factors controlling the spatial patterns of N cycling in these soils, as well as factors that potentially predispose forest soils to N saturation.

## EXPERIMENTAL METHODS/PROCEDURES

### Study Site

Fernow Experimental Forest occupies (FEF) approximately 1,900 ha of the Allegheny Mountain section of the unglaciated Allegheny Plateau in Tucker County, WV (39°03′N, 79°49′W). Precipitation at FEF averages approximately 1,430 mm year$^{-1}$, generally increasing during the growing season and with elevation[10].

Two watersheds served as study sites: WS4 (>80-year-old mixed-aged stand) and WS3 (~30-year-old even-aged stand). WS3 served as the "treatment" watershed, whereas WS4 was the control. Beginning in 1989, WS3 has received three aerial applications of $(NH_4)_2SO_4$ year$^{-1}$, totaling 35 kg N ha$^{-1}$ year$^{-1}$. The total amount of N deposited on WS3 is approximately 54 kg N ha$^{-1}$ year$^{-1}$ (application plus atmospheric deposition)[11]. Soils are similar between the two study watersheds. These are largely coarse-textured Inceptisols (loamy-skeletal, mixed mesic Typic Dystrochrept) of the Berks and Calvin series, sandy loams derived from sandstone[6].

Early-successional tree species, such as black birch, black cherry, and yellow poplar, dominated stands on WS3, whereas late-successional species, such as sugar maple and northern red oak, dominated in stands on WS4. Dominant herbaceous-layer species were similar between watersheds and included stinging nettle, violets, and several fern species[12].

### Field Sampling

Field sampling resembled a 2 × 2 factorial design, with two N treatment levels (N-treated WS3 vs. untreated WS4) and two rates of N processing (high vs. low). Thus, sampling was done in four plots total: one high-rate plot and one low-rate plot in each of the two watersheds. Designation of high vs. low rates were determined from a combination of 3-year means of concentrations of soil water $NO_3^-$ and 3-year means of rates of net N mineralization and nitrification (Fig. 1).

Forest floor material (O1 horizon) and mineral soil samples were taken (5-cm depth) from three randomly located subplots within each plot. Surgical gloves and trowels were used for soil sampling and were washed with an antibacterial solution between subplots within each plot and between plots to eliminate contamination between samples.

### Laboratory Analyses

All soil samples were brought back to the laboratory at Marshall University for initial characterization of the microbial community and determination of preincubation levels of $NH_4^+$ and $NO_3^-$. Each soil sample was divided into three subsamples, with each group of subsamples being incubated at one of three temperatures: 10, 20, and 30°C. All subsamples were extracted following incubation for 28 d. Extraction and analysis for $NH_4^+$ and $NO_3^-$ were done for mineral soil only and followed methods described in Gilliam and Adams[13]. Briefly, moist soils were extracted with 1 N KCl at an extract to soil ratio of 10:1 (v:w). Extracts were analyzed colorimetrically for $NH_4^+$ and $NO_3^-$ with a Bran+Luebbe TrAAcs 2000 automatic analysis system. Net N mineralization was calculated as postincubation ($NH_4^+$ plus $NO_3^-$) minus preincubation ($NH_4^+$ plus $NO_3^-$); net nitrification was calculated as postincubation $NO_3^-$ minus preincubation $NO_3^-$.

Forest floor samples were oven dried (50°C) and ground via Wiley Mill to pass a 40-mesh screen. Ground samples were analyzed for total N with a Bran+Luebbe TrAAcs 2000 auto-

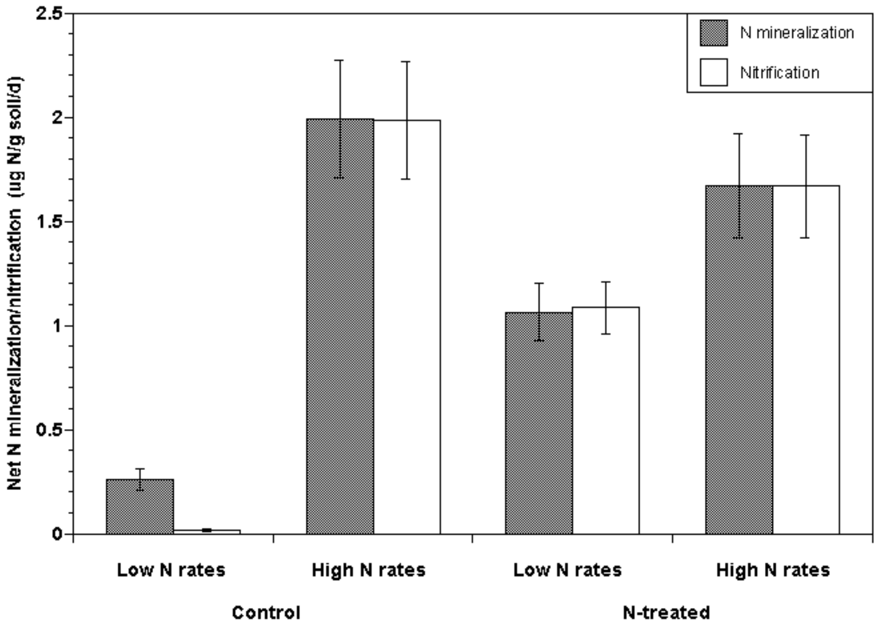

**FIGURE 1.** Three-year mean net N mineralization and nitrification rates for plots from two watersheds (control vs. N-treated) of Fernow Experimental Forest, West Virginia, with contrasting rates of soil N processing (low N rates vs. high N rates). Error bars are given as ±1 SE of the mean.

matic analysis system following block digestion, and for lignin using the method of Allen[14].

Preincubation extracts were analyzed with a Varian inductively coupled plasma emission spectrophotometer for Mg, Ca, and Al. Soil pH was determined with a 10:1 (v:w) ratio of 1 *N* KCl to moist soil using a 720A pH/ISE meter and Ross combination electrode.

## PCR Primers

Thirty-nine ammonia monooxygenase subunit A (*amo*A) gene sequences were recovered from GenBank (www.ncbi.nlm.nih.gov) and aligned using ClustalX (the ClustalX program can be obtained at ftp://ftp-igbmc.u-strasbg.fr/pub/ClustalX/). The aligned sequences were inspected for conserved regions that could be used as sites for primer annealing. No priming sites were found to be conserved throughout all of the known *amo*A sequences. Therefore, the aligned sequences were used to generate a phylogenetic tree using the Neighbor-Joining option of ClustalX. The tree file was viewed using TreeView (TreeView is available at http://taxonomy.zoology.gla.ac.uk/rod/treeview.html), and three phylogenetically coherent *amo*A subgroups were chosen as amplification targets. Sequences within these *amo*A subgroups were aligned and inspected for conserved regions. Putative primer sequences were checked for potential primer dimer formation and intrastrand base-pairing using Primers! Lite. Acceptable primer sequences were synthesized at the Marshall University Core DNA facility. Table 1 shows the primer sequences that correspond to each of the three *amo*A subgroups.

## Isolation and Purification of DNA

Site nucleic acids were isolated and purified using the direct lysis method of Ogram[15]. Soil samples (5 g) were washed twice with sodium phosphate buffer to remove cell-free nucleic acids. Washed soils were treated with lysozyme, and microbial cells were lysed by repeated freezing and thawing. The crude lysate was partially purified by treatment with NaCl and hexadecyltrimethyl ammonium bromide (CTAB) followed by extraction with chloroform:isoamyl alcohol (24:1). Polyethylene glycol (PEG) was used to precipitate nucleic acids from the aqueous phase. The nucleic acids were resolubilized in Tris-EDTA (TE) buffer and reprecipitated with ammonium acetate and ethanol.

One additional treatment with NaCl, CTAB, and chloroform:isoamyl alcohol followed by ethanol precipitation was required to obtain amplifiable DNA. Nucleic acids were solubilized in TE buffer for use in amplification reactions.

## Amplification

Ammonia monooxygenase genes were amplified from purified site DNA using a PCR Core Kit (Boehringer-Mannheim, Indianapolis, IN) according to the manufacturer's instructions. The final volume of the reaction mixtures was 100 µL.

The thermal program for amplification included four cycles. Cycle 1 consisted of a single step at 94°C for 5 min. *Taq* DNA polymerase was added to the amplification reactions during cycle one. Cycle 1 was not repeated. Cycle 2 consisted of three steps: 94°C for 1 min., 55°C (initial) for 2 min., and 72°C for 3 min. Cycle 2 was repeated 18 times, during which the annealing temperature was decreased from 55 to 37°C. All other parameters of

**TABLE 1**
**Amplification Primers and the Sources of *amo*A Genes with Complementary Binding Sites**

| Primer Sequences | Sources of *amo*AGene Sequences (GenBank Accession Number) |
|---|---|
| 5′-CGC AAC TGG ATG ATC AC –3′ (forward) | *Nitrosospira briensis* C-128 (U76553) |
| 5′-ACC AGC GGC AGG TGG GTC-3′ (reverse) | *Nitrosospira briensis* C-128 (Z97859) |
| | *Nitrosospira briensis* C-57 (Z97858) |
| | *Nitrosospira multiformis* C-71 (X90822) |
| | *Nitrosospira multiformis* Nm24C (AF042171) |
| | *Nitrosospira* sp. AHB1 (X90821) |
| | *Nitrosospira* sp. Np 39-19 (AF006692) |
| | *Nitrosospira* sp. Np 39-19 (AF016002) |
| | *Nitrosospira tenuis* Nv-1 (Z97860) |
| | *Nitrosovibrio tenuis* Nv-12 (U76552) |
| | Environmental clone RR 45-2 (Z97833) |
| | Environmental clone RR 90-6 (Z97839) |
| | Environmental clone RR 90-7 (Z97840) |
| | Environmental clone SP-14 (Z97849) |
| 5′-TAC CCC ATC AAC TTT GTA TTC CC-3′ (forward) | Environmental clone RR 45-3 (Z97834) |
| 5′-GCC AAA TGC GGT GAC GTC GTT CT-3′ (reverse) | Environmental clone RR 45-7 (Z97836) |
| | Environmental clone RR 90-8 (Z97841) |
| | Environmental clone RR 90-9 (Z97842) |
| | Environmental clone RR 90-14 (Z97843) |
| | Environmental clone RR 90-17 (Z97844) |
| 5′-TTT GTA CTG CCA TCG ACC ATG AT-3′ (forward) | Environmental clone Schohsee (Z97851) |
| 5′-CGC AGA ACA TCA GCA TAG ATA C-3′ (reverse) | Environmental clone SP-9 (Z97848) |

cycle 2 remained unchanged. Cycle 3 consisted of three steps: 94°C for 1 min., 52°C for 2 min., and 72°C for 3 min. Cycle 3 was repeated 30 times. Cycle 4 consisted of three steps: 94°C for 1 min., 52°C for 2 min., and 72°C for 7 min. Cycle 4 was not repeated. Completed amplification reactions were stored at –20°C prior to analysis by agarose gel electrophoresis.

### *Agarose Gel Electrophoresis*

Ammonia monooxygenase amplification reactions were analyzed by agarose gel electrophoresis using a mini-gel format (Bio-Rad Mini-Sub Cell GT, Bio-Rad Laboratories, Hercules, CA). Agarose (1.5%; Bio-Rad Molecular Biology Certified) was dissolved in Tris-acetate-EDTA (TAE) buffer (40 m*M* Tris-acetate, 1 m*M* EDTA [pH 8.0]) by heating to approximately 95°C for 1 min. The agarose solution was cooled to 48°C, and ethidium bromide (0.2 µg $ml^{-1}$) was added prior to pouring. Each gel was loaded with up to seven amplification reactions and one DNA size standard (1 Kb ladder; Promega, Madison, WI). DNA fragments were resolved in TAE buffer at 100 V for 45 min. Amplicons were visualized by ultraviolet illumination and photographed with a Polaroid camera equipped with a Tiffen® Deep Yellow 15 filter and Polaroid 667 film.

### Data Analyses

Means of all analyzed variables, including (1) net N mineralization and nitrification at 10, 20, and 30°C; (2) litter N, lignin, and lignin to N ratio; and (3) soil pH and extractable Mg, Ca, Al, and Ca to Al ratios, were compared across all four watershed and N status combinations using one-way analysis of variance and Bonferroni multiple-range tests[16]. Means of net N mineralization and nitrification were compared across the three incubation temperatures for each watershed/N status combination separately, also using one-way analysis of variance and Bonferroni multiple-range tests[16].

To test for variables that best predict net nitrification in these soils, *a posteriori* comparisons (linear and curvilinear) were performed for plot means of nitrification rates at each incubation temperature and compared to plot means of all other analyzed variables to find the best fit (i.e., highest $r^2$).

## RESULTS AND DISCUSSION

### Soil and Litter

Results of this study confirm spatial patterns of net N mineralization and nitrification observed in the field, as reported in

Gilliam et al.[9] and summarized in Fig. 1. That is, net changes in inorganic soil N under these controlled-temperature incubations exhibited contrasts among plots that were similar to those found for *in situ* field incubations, especially for nitrification. These rates were in the following order: control/high N > N-treated/high N > N-treated/low N > control/low N (Table 2). Also consistent with earlier results, the control/low N plot exhibited essentially no net nitrification throughout the 28-d incubation period, regardless of temperature. Extractable N pools also exhibited these contrasts among plots, with the exception that extractable $NH_4^+$ for control/low N was the same as that for N-treated/low N (Table 3).

Net N mineralization exhibited a sensitive response to incubation temperature, with highest mineralization rates occurring at 30°C for all plots; for the three plots wherein net nitrification did occur, nitrification responded to temperature in a way that was similar to that for mineralization (Table 2). In both low N plots, net N mineralization increased linearly with temperature, whereas it increased exponentially with temperature in both high N plots. This was also the case for net nitrification (except for in the control/low N plot) (Table 2).

Stark[17] also found curvilinear increases of nitrification in response to increasing incubation temperatures up to 30°C (beyond which there was a significant decline) for N-amended soils from a California oak woodland–annual grassland site, determining that temperature optima for grassland and oak woodland soils were 35.9 and 31.8°C, respectively. Similarities in response of nitrification to temperature between soils of the California site[17] and FEF soils suggest that nitrifier populations at FEF may also have temperature optima at >30°C. This is well above the highest mean daily temperature (20.4°C) during a single growing season reported by Gilliam and Adams[13] for undisturbed surface soils at another FEF site close to these study watersheds (mean [±1 SE] for entire growing season = 15.7 ± 0.4°C; median = 16.7°C). Furthermore, it is higher than the highest mean daily temperature (28.9°C) during the same period for surface soils following whole-tree harvesting at FEF (mean

**TABLE 2**
**Means (±1 SE) for Net N Mineralization and Nitrification at Three Incubation Temperatures for Low N Processing and High N Processing Plots Within Control and N-Treated Watersheds of Fernow Experimental Forest, West Virginia***

| | N Mineralization | | | Nitrification | | |
|---|---|---|---|---|---|---|
| | µg N g$^{-1}$ soil d$^{-1}$ | | | | | |
| Treatment/N Status | 10°C | 20°C | 30°C | 10°C | 20°C | 30°C |
| Control/low N | $0.16 \pm 0.08_y^b$ | $0.61 \pm 0.15_{xy}^c$ | $1.22 \pm 0.28_x^b$ | $0.01 \pm 0.01_x^c$ | $0.01 \pm 0.01_x^c$ | $0.01 \pm 0.01_x^c$ |
| Control/high N | $1.17 \pm 0.18_y^a$ | $2.97 \pm 0.43_y^a$ | $9.43 \pm 1.67_x^a$ | $0.83 \pm 0.18_y^a$ | $2.69 \pm 0.39_y^a$ | $8.90 \pm 1.45_x^a$ |
| N-treated/low N | $0.32 \pm 0.06_y^b$ | $0.67 \pm 0.14_{xy}^c$ | $1.18 \pm 0.21_x^b$ | $0.24 \pm 0.03_y^b$ | $0.56 \pm 0.14_{xy}^c$ | $0.94 \pm 0.19_x^c$ |
| N-treated/high N | $0.91 \pm 0.29_y^a$ | $1.81 \pm 0.44_{xy}^b$ | $3.64 \pm 0.88_x^b$ | $0.74 \pm 0.31_y^a$ | $1.57 \pm 0.47_{xy}^b$ | $3.50 \pm 0.92_x^b$ |

* Means for a given variable followed by the same superscript (a, b, c) are not significantly different between plot types at $p < 0.10$. For plot types, means for net nitrification followed by the same subscript (x, y) are not significantly different between incubation temperatures at $p < 0.10$.

**TABLE 3**
**Means (±1 SE) for Extractable $NH_4$ and $NO_3$ Pools, Litter Lignin and N Concentrations, and Lignin to N Ratios for Low N Processing and High N Processing Plots Within Control and N-Treated Watersheds of Fernow Experimental Forest, West Virginia***

| Treatment/N Status | $NH_4$ | $NO_3$ | Lignin % | N % | Lignin to N |
|---|---|---|---|---|---|
| | µg N g$^{-1}$ soil | | | | |
| Control/low N | $2.6 \pm 0.3^b$ | $0.3 \pm 0.1^c$ | $32.8 \pm 0.8^a$ | $1.44 \pm 0.11^b$ | $22.9 \pm 1.4^a$ |
| Control/high N | $4.4 \pm 0.6^a$ | $5.4 \pm 1.7^a$ | $33.1 \pm 0.6^a$ | $1.73 \pm 0.03^a$ | $19.1 \pm 0.3^b$ |
| N-treated/low N | $2.6 \pm 0.1^b$ | $1.3 \pm 0.8^{bc}$ | $35.9 \pm 0.7^a$ | $1.82 \pm 0.03^a$ | $19.8 \pm 0.7^{ab}$ |
| N-treated/high N | $3.4 \pm 0.4^{ab}$ | $3.1 \pm 0.7^{ab}$ | $33.8 \pm 1.1^a$ | $1.97 \pm 0.02^a$ | $17.2 \pm 0.6^b$ |

* Means for a given variable followed by the same superscript are not significantly different between plot types at $p < 0.10$.

[±1 SE] for entire growing season = 20.5 ± 0.7°C; median = 20.9°C)[13].

Lignin content of forest litter did not vary significantly among plots; by contrast, litter N was significantly lower for the control/low N plot than for the other plots (Table 3). Consequently, lignin to N ratio in forest litter, often a good predictor of N mineralization in forest soils[18,19], was significantly highest for the control/low N plot (Table 3).

All soils were acidic, with individual subplot pH values ranging from 3.30 to 4.15, below those typically associated with measurable rates of net nitrification[20]. Lowest pH values were found for the low N plots, whereas highest pH was found in the control/high N plot (Table 4). Although soil Mg did not vary significantly among plots, Ca was significantly highest in the high N plots and lowest in the control/low N plot. The opposite pattern was apparent for soil Al, with the low N soil having higher concentrations of Al than the high N soil. Ratios of Ca to Al, which have been used as indicators of environmental stress in forest ecosystems[21,22], varied significantly (and by over an order of magnitude) among plots, from just under 1.00 for the control/high N plot to <0.10 for both low N plots (Table 4).

## Microbial Community

The oligomer primer sets shown in Table 1 were designed to detect the presence of *amo*A genes in FEF soils. Ammonia monooxygenase catalyzes the first step in nitrification, and can therefore be used as an indicator of nitrification potential. The three primer sets used herein detect approximately half of the known *amo*A genes. The results of amplification with these primers are shown in Table 5. The genetic potential for ammonia oxidation was demonstrated in three of the four plots under study. Most notably, the control/low N plot was found to have two different types of ammonia monooxygenase genes present. This means that the genetic potential for ammonia oxidation was present, even though nitrification was not observed. Therefore, the lack of nitrification observed both in the field (Fig. 1) and under controlled incubations (Table 2) for the control/low N plot cannot be explained by an absence of a nitrifying microbial community, and we must reject our original hypothesis.

It should also be noted that *amo*A was not detected in the N-treated/high N plot even though nitrification was demonstrated in this plot. This is due to the fact that not all *amo*A sequences in nature are known, and that these three primer sets do not detect

**TABLE 4**
**Means (±1 SE) for Extractable Mg, Ca, and Al; pH; and Ca to Al Ratios for Low N Processing and High N Processing Plots Within Control and N-Treated Watersheds of Fernow Experimental Forest, West Virginia***

| Treatment/ N Status | pH | Mg | Ca | Al | Ca to Al |
|---|---|---|---|---|---|
| | | μg g$^{-1}$ soil | | | |
| Control/low N | 3.47 ± 0.10$^{c}$ | 1.5 ± 0.6$^{a}$ | 21.9 ± 7.1$^{c}$ | 712.9 ± 120.6$^{a}$ | 0.04 ± 0.02$^{b}$ |
| Control/high N | 4.00 ± 0.08$^{a}$ | 3.4 ± 3.4$^{a}$ | 86.8 ± 43.6$^{a}$ | 149.0 ± 102.1$^{c}$ | 0.77 ± 0.32$^{a}$ |
| N-treated/low N | 3.46 ± 0.04$^{c}$ | 0.6 ± 0.6$^{a}$ | 33.4 ± 6.6$^{ab}$ | 520.7 ± 34.0$^{ab}$ | 0.06 ± 0.01$^{b}$ |
| N-treated/high N | 3.75 ± 0.05$^{b}$ | 1.4 ± 0.6$^{a}$ | 93.1 ± 19.7$^{a}$ | 293.4 ± 30.3$^{bc}$ | 0.32 ± 0.08$^{ab}$ |

* Means for a given variable followed by the same superscript are not significantly different between plot types at $p < 0.10$.

**TABLE 5**
**Presence of Ammonia Monooxygenase Enzyme Types in Low N Processing and High N Processing Plots Within Control and N-treated Watersheds of Fernow Experimental Forest, West Virginia***

| | Control | | N-treated | |
|---|---|---|---|---|
| Enzyme Type | Low N | High H | Low N | High N |
| *Nitrosospira briensis* C-128 | + | + | + | – |
| Environmental clone RR 45-3 | – | – | – | – |
| Environmental clone Schohsee | + | + | – | – |

* A "+" indicates presence, whereas a "–" indicates absence.

all known *amo*A sequences. Nitrification at the N-treated/high N plot did not involve an *amo*A gene that was detected with these primers.

## Factors Influencing Net Nitrification

One of the more serious concerns associated with N-saturated soils is their tendency to leach $NO_3^-$along with essential base cations[8,23,24], potentially leading to soil cation depletion and representing a challenge to sustainable management of N-saturated forests[13,25,26,27,28]. Accordingly, a major focus of this study was on the spatial patterns of net nitrification at FEF, and to determine which forest floor and soil factors best explain those patterns. Because the four plots chosen for this study represent the full range of rates of net nitrification reported for FEF[9], it is a reasonable assumption that quantitative comparisons between plot means of nitrification rates and corresponding forest floor and soil variables should provide a reliable assessment.

Surprisingly, none of the forest floor variables were correlated with net nitrification, including lignin content, N content, and lignin to N ratios (Table 6). This lack of correlation could be related in part to the low range of values found in our study. For example, our values for lignin to N ratio ranged from ~17 to 23. Studies of conifer stands of the Rocky Mountains[18,19] found highly significant relationships between lignin to N ratios and net N mineralization, but they also reported ranges of lignin to N from less than 20 to greater than 70, with net N mineralization occurring at lignin to N of <50. Their highest rates of mineralization occurred at lignin to N of ~20, close to the value of our plot exhibiting no net nitrification. Clearly, forest floor lignin to N does not provide an adequate explanation for the lack of net nitrification in the control/low N plot.

Although extractable Mg was also not correlated with net nitrification, Ca, Al, and pH were all significantly correlated ($p < 0.10$) with nitrification for at least one of the three incubation temperatures (Table 6). These correlations, however, assume a linear relationship between variables. Closer inspection of the data revealed that some of these relationships were curvilinear. Indeed, the best fit (i.e., highest $r^2$) was found for net nitrification vs. soil Ca to Al ratio using logarithmic models (Fig. 2). Thus, it appears that the relative balance of Ca and Al in the soil explains the spatial pattern of net nitrification at FEF, rather than variations of either Ca or Al alone. Soil pH, which is closely connected with the Ca/Al balance in many soils[20], also did a good job explaining the spatial patterns of net nitrification, exhibiting a significantly positive linear relationship with $r^2$ values of 0.85, 0.96, and 0.96 for incubation temperatures of 10, 20, and 30°C, respectively.

Ratios of Ca to Al in soil have been used as indicators of forest health. Although no set criteria are widely accepted, Cronan and Grigal[21] made a strong argument for a series of ratio values for (1) soil solution, (2) fine root, and (3) foliar tissue as indications of stress in forest ecosystems. More recently, Demchik and Sharpe[22] reported soil-extractable Ca to Al ratios in northern red oak stands that were comparable to those found in our high N plots (Table 4). Our data suggest that generalizations regarding the ecological significance of Ca to Al ratios might not be directly applicable to N-saturated systems, considering that high ratios at FEF likely result in the highest level of $NO_3^-$ leaching.

## CONCLUSION

This study has demonstrated that soil chemistry may exert a more profound influence on soil N processing than is often discussed in the literature, wherein litter chemistry (particularly lignin to N ratio) usually has been shown to control rates of net N mineralization and nitrification[18,19]. Our range of values for such rates was quite wide (e.g., nitrification ranged from 0.01 to 8.9 µg $NO_3^-$-N $g^{-1}$ soil $d^{-1}$ at 30°C), whereas our range for litter chemical characteristics clearly was not.

One of the main objectives of this study was to investigate possible mechanisms that would explain the unusual occurrence of soils that exhibited little, if any, detectable net nitrification within WS4, a watershed that has exhibited elevated stream losses of $NO_3^-$-N over the past several decades[26]. The most parsimonious hypothesis—the one tested in this study—is that these soils lack nitrifier populations. Using qualitative microbial techniques, we have found evidence to reject this hypothesis. We must, therefore, conclude that nitrifier populations are present in these soils but are being inhibited by some factor, or perhaps a suite of factors.

Gilliam et al.[9] suggested that presence of ericaceous species in the herbaceous layer may be a factor limiting nitrification on WS4. They found that hillside blueberry (*Vaccinium vacillans*)

**TABLE 6**
**Linear (Pearson Product-Moment) Correlations of Forest Floor and Soil Variables with Net Nitrification at Three Incubation Temperatures (10, 20, 30°C)***

| Net Nitrification at: | Lignin | N | Lignin to N | Mg | Ca | Al | Ca to Al | pH |
|---|---|---|---|---|---|---|---|---|
| 10°C | NS | NS | NS | NS | 0.98 | –0.99 | NS | 0.92 |
| 20°C | NS | NS | NS | NS | NS | –0.98 | 0.97 | 0.98 |
| 30°C | NS | NS | NS | NS | NS | –0.92 | 0.99 | 0.98 |

* Lignin, N, and lignin to N ratio data are of forest floor material. Other data are of soil extracts. Values shown are correlation coefficients (r) significant at $p < 0.10$; n = 4 for all correlations. NS indicates correlation not significant at $p < 0.10$.

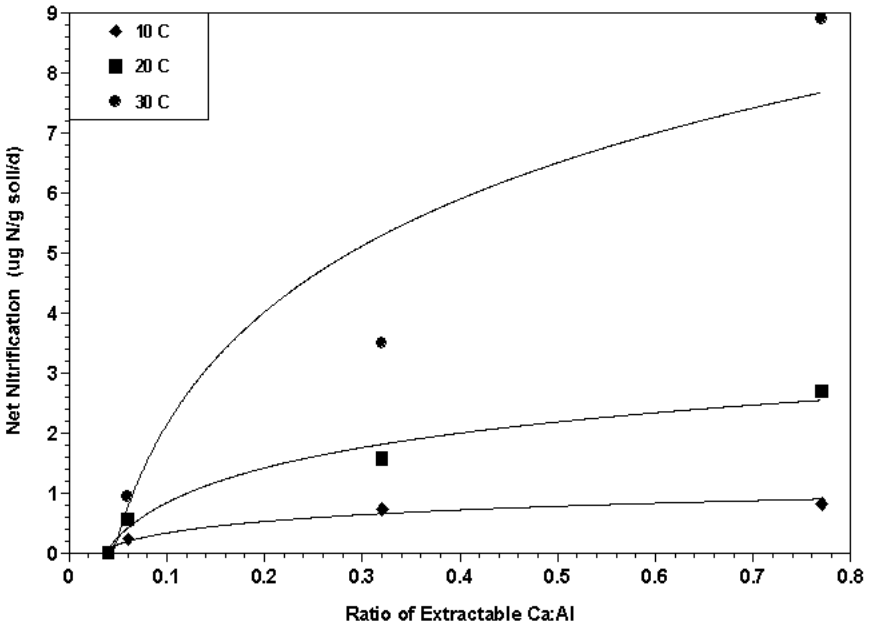

**FIGURE 2.** Net nitrification vs. ratio of extractable Ca to Al among four plots of contrasting N treatment and rates of N processing at three incubation temperatures: 10, 20, and 30°C. Equations (all significant at $p < 0.05$) for the three incubation temperatures were, respectively: $y = 0.97 + 0.28 \ln(x)$, $r^2 = 0.96$; $y = 2.76 + 0.84 \ln(x)$, $r^2 = 0.97$; $y = 8.38 + 2.71 \ln(x)$, $r^2 = 0.90$.

was nearly 30% of herb layer above-ground biomass within the area of WS4 exhibiting no net nitrification. Neither this nor any other ericaceous species were found on the other plots sampled within WS4. Ericaceous species are known to maintain, and even increase, soil acidity via preferential uptake of $NH_4^+$ over $NO_3^-$[29]. Perhaps more important, roots of ericaceous species support ericoid mycorrhizae that are capable of secreting organic acids that limit N-mineralizing microbes and, particularly, nitrifying bacteria[30].

In addition to this possible allelopathic inhibition, data from the present study suggest that there may be a strong inhibitory effect of soil chemistry (especially Ca to Al ratio) on the activity of nitrifying bacteria, considering that the control/low N plot exhibited evidence of the presence of nitrifier populations, but not of net nitrification. We are unaware of studies that have examined the direct effects of Ca to Al ratios on soil nitrifier populations. Thus, this is an area worthy of further investigation.

From a practical viewpoint, if Ca to Al ratios do limit soil nitrifier populations in ways suggested in this study, then such a limitation poses a serious challenge to the management of N-saturated forests, wherein lime treatments (e.g., applications of dolomite or calcite limestone) are indicated to alleviate problems associated with cation loss. That is, additions of Ca via lime may further increase already high rates of net nitrification. Watershed-scale additions of predominantly calcite limestone resulted in significant increases in nitrification rates in Adirondack Park, New York[31]. These challenges to forest management are further complicated by the species-specific and site-specific (e.g., mesic vs. xeric) variation in Ca processing in hardwood forests[32].

| | | |
|---|---|---|
| Received: | July | 23, 2001 |
| Revised: | September | 10, 2001 |
| Accepted: | September | 10, 2001 |
| Published: | October | 17, 2001 |
